# The Possibilities of the Callahan Method

**Published:** 1921-05

**Authors:** Harry B. Johnston


### THE POSSIBILITIES OF THE CALLAHAN METHOD

BY HARRY B. JOHNSTON, D.D.S.

Permit me to say at the outset that I have studied in detail and extensively experimented with a great many methods of canal opening and filling and have chosen the Callahan method because of immeasurably superior results obtained, particularly so since adopting some slight modifications of, and additions to, the original technic.

It is my contention that with the Callahan method it is not only possible, but easy to fill and hermetically seal canals heretofore considered impossible.

Briefly stated, the Callahan method consists of the use of dilute sulphuric acid as a decalcifying medium to assist in opening the canals, followed by a saturate solution of sodium bicarbonate, causing a rapid effervescence which effectively clears out all debris. The acid is also a powerful though superficial germicide. After dehydration the canal is flooded with a solution of chloroform and fiddle-bow resin in the proportion of twelve grains of resin to three fluid drams of chloroform. A point of pure base plate gutta-percha, only slightly smaller in diameter, but longer than the canal, is then selected, passed into the canal and caused to dissolve in the chloroform by a rapid though delicate pumping or stirring motion. We will now take this up step by step and study the ''what'' and ''how'' of it.

### SULPHURIC ACID AS A CLEANSING AGENT

For this purpose 30 to 50 per cent. aqueous solution of the chemically pure acid is used. It softens the wall of the canal by a process of decalcification so as to permit easy and fairly rapid enlargement of its caliber by curetting with various instruments. The most valuable factor in the use of the acid, however, is that it may safely be sealed in the canal, thereby giving it time to diffuse through accessory canals which can not be reached with any instrument and can be treated *only* by grace of the law of diffusion of liquids. It must always be borne in mind that our only real problem is to *fill these accessory canals and seal their foramina.* By diffusion, the sulphuric will penetrate these more safely than any other chemical now at our disposal. It is far from being as effective in dissolving the organic content of these canaliculi as we would like, but so far the prime factor of safety to the patient has prevented the sealing in of any more effective solvent; for bear in mind that these canaliculi can be reached *only* by diffusion, which requires time. The organic matter in the canals and canaliculi is attacked to a marked degree, caused to yield up its water, shrink and release its hold upon the tube wall. The effervescence caused by the addition of the soda will then break it up and remove it cleanly from the larger canals and to a considerable extent from the smaller canaliculi. But although the organic matter is not perfectly removed from these, part of that remaining is forced out of the foramina into the tissues where it is promptly digested and the remainder encapsulated in the filling.

The advantages of dilute sulphuric acid then appear to be as follows: (1) It permits of the rapid and perfect cleaning and enlarging of the main canal. (2) Its marked affinity for water causes its rapid diffusion through all the accessory canals where its well-known oxidizing properties have a burning or charring effect upon dead tissue. (3) The addition of soda produces such a violent effervescence that not only this debris, but frequently even broken instrument points will be forced out by it. (4) Last, but by no means least, it may be safely sealed in the tooth, which will allow ample time for this diffusion through all canals and canaliculi, enlarging them by a self-limiting decalcification and cleaning and sterilizing by oxidation.

We might as well settle the question, "Are you not afraid you will get some of the acid through the apex?" Yes, I am, and you sometimes do, even if your technic is the most careful, but all of our present methods are so imperfect in one point or another that no man can reasonably expect to get perfect results in every case by any method. With reasonable care, however, this result would be the very great exception; for bear in mind that any liquid you place in the canal does not go ahead of the instrument, but follows it, and you are much more liable to get a laceration of the periapical tissue from your instrument point than an irritation from the acid. In a root having a large foramen you have only slight need for the acid, and it, like any other similar agent, must be cautiously and sparingly used in this type canal. It should not and need not be sealed in a canal of this kind.

### TECHNIC AND INSTRUMENTATION

After the canal has been freed of pulp tissue, or old filling, the crown of the tooth is coated with cavity varnish, sandarac, or chloro-resin, the pulp chamber flooded with 50 per cent. acid and the canal entered with a fine or extra fine Kerr pulp canal file. This is gently passed in until it hangs. It is then given about one-eighth twist, held in that position, and withdrawn. This is repeated, pumping the acid in thoroughly until the instrument and acid have penetrated to about the apical third or has gone as far as it will easily go. The next smaller size file is then used and the opening and pumping continued slightly farther. Measuring wires may be used at this point if desired. No instrument larger than the finest smooth broach or pathfinder should ever be passed through the foramen if it can be avoided. In the majority of cases it is easy to tell when you are about to enter the foreman, as there is a slight constriction just short of the actual opening. The acid should be thoroughly pumped to this point and neither acid nor instrument carried any farther. It is difficult to coax the acid to this point and there is small danger of getting it any farther, except, of course, in the very large canals. The acid is neutralized at frequent intervals removing the debris, and just before sealing the cavity and dismissing the patient, fresh acid is pumped into the canal, the surplus absorbed out of the pulp chamber, a pellet of sterile cotton placed in, and the cavity sealed with hot temporary stopping without pressure. This may be left in for any length of time that may be desired or necessary, as the acid is self-limiting in its action and, due to the alkalinity of the lime salts of the dentin, does not remain active for more than two or three hours.

If the canal in which you are working is not perfectly straight and your Kerr file hangs at some point, or suddenly fails to penetrate farther, remove it from the canal, and with your radiograph as a guide, bend the point of the file slightly, reintroduce into the canal, and probe for the opening into the remainder of the canal. The very great majority of canals can be freely and easily opened if we can bend our file to approximate the curve of the canal. Force plays no part in the opening of a pulp canal—it must be done by "persuasion."

Let us study for a few moments just what the acid does while it is in the canal and how it does it. In a pulpless tooth all of the canals and canaliculi are as a rule filled with decomposed organic matter in aqueous solution. That can be removed from the main canal by absorption, but it can not be so removed from the fine accessory canals. When the main canal is flooded with the acid, then the law of the diffusion of liquids comes to our help, and as the 50 per cent. of water contained in the acid solution is not sufficient to satisfy the acid, it takes up more water from whatever source it can gather it. Sealed in the tooth, its only available supply is contained in the accessory canals and to some extent in the tubuli. It can gather but little from the tubuli, however, because of the wall of calcium sulphate which it quickly builds up around itself and which prevents its further penetration. It must therefore act upon the watery content of the accessory canals, which it promptly does. The water in these canals is drawn into the acid and the acid flows back into the canals. In other words, sufficient of the water and the acid change places to produce a balancing of the solution. Does any of this acid diffuse into the tissues beyond the foramen? Not unless the apical tissue has been broken down and replaced with cystic fluid. Its action in coagulating albumen will not permit it to actually enter living tissue. There again it is self-limiting. It must have a fluid medium, and finding such in the accessory canals it follows them to the ends, oxidizes and breaks up the dead tissues contained in them, and destroys the bacteria. In treating the canal it is not possible to ascertain where these accessory canals are, their number, nor direction; but if they are present (and we are told by excellent authorities that they always are) the acid will find them.

Subsequent application of fresh acid, followed by the soda, will result in the complete removal of all organic material from all of these canals excepting those exceedingly small and quite longer than the average.

It might be well at this point to explain the part that capillary attraction plays in this process. Capillary attraction has practically nothing to do with it, and what little part it does play is confined entirely to the main canal.

There are two kinds of capillary attraction which I will for convenience call primary and secondary. Primary capillary attraction is that attractive force exercised by the walls of a glass or other non-porous capillary tube. If this type of capillary has one end closed either by fusing, by placing a minute quantity of liquid in it, or being surrounded by the periapical tissues, as is the case with the root capillary, its force of attraction is completely broken. Therefore there is no such thing as capillary attraction of the primary type in the root-canal of a tooth. That which I have called ''secondary capillary attraction'' is exercised by any porous substance capable of absorbing water or watery solutions, and its force is neutralized when the microscopic capillaries of the substance become filled with the solution. Therefore it will be seen that if you can dehydrate the walls of the canal and accessory canals you can put this type of attraction in force. This, however, can not be done in the accessory canals, and so these canals (or canaliculi) which constitute our great problem in pulpcanal filling can be reached, cleaned and filled only by diffusion. In other words, you can only get one substance out of them by substituting another.

### FILLING THE CANALICULI BY DIFFUSION

When we have finished with our acid and neutralized it for the last time, our canaliculi are filled with water, holding any chemical compounds in solution. The water effectively bars the entrance of our chloro-resin solution, so we must substitute something else for this water, something which has an affinity for chloroform. Alcohol is ideal for this purpose. The main canal is then flooded with 95 per cent. alcohol, which is thoroughly pumped and churned into it. By diffusion this substitutes for the water in the canaliculi, filling them with a lower-grade alcohol. Ample time must be allowed for this, for if the canaliculi contain less than 85 per cent. alcohol, the chloro-resin will not diffuse through them. This usually requires from three to five minutes. All of the alcohol which can be removed with absorbent points is then taken out and the pulp chamber and canal flooded with chloro-resin. No pulp-canal driers or hot air must be used at this stage, for we wish the canaliculi to remain filled with alcohol. The chloroform diffuses perfectly and rapidly into the alcohol, and inasmuch as the resin is in perfect solution in the chloroform, wherever the chloroform goes the resin goes. Time is required here also. It is not a ''slam-bang'' process. Allow from three to five minutes here, pumping in fresh chloro-resin as the chloroform is soaked up by the tubuli and canaliculi or passes off by evaporation. Toward the end of this period, when the solution in the canal begins to get rather heavy, add pure chloroform as needed to keep it sufficiently thin to dissolve the gutta-percha point.

When this third substitution or diffusion is complete, a gutta-percha point of suitable size and shape is then passed into the canal and dissolved by either a pumping or stirring movement, which movement you use is governed by the size of the foramen. With a normal, small foramen, a pumping movement is best; with a large foramen this would force too much chloro-percha through, therefore a stirring movement is used.

Just how beautifully the gutta-percha will diffuse through the chloroform is shown by the illustrations of decalcified teeth and radiographs of teeth filled in the mouth.

We are told, and correctly I think, that chloro-percha in hardening shrinks from the periphery. In other words, it releases the walls of its container. And we are also told that chloro-resin-percha in hardening shrinks from the center, or clings to the wall of its container by virtue of the strong adhesion of the resin and the fact that tiny threads of this material actually enter the tubuli. An excellent way to assist this central shrinkage and peripheral adhesion is as follows: After the filling has been sufficiently condensed with the canal pluggers, select a plugger of suitable size, and by passing it down through the center of the filling for at least half the length of the canal entirely break up all central cohesion.

The pulp chamber should then be quickly filled with cement so as to prevent external evaporation of the chloroform, for if the chloroform is permitted to evaporate externally, even the above precaution will not prevent peripheral shrinkage.

Nothing has been said so far about the effectiveness of the resin in filling the tubuli. Chloroform is one of the most penetrating liquids we have, and it not only quickly penetrates the tubuli, *but the inter-prismatic spaces* between the enamel rods as well. This can be easily proved. Dehydrate an extracted tooth either by allowing it to dry out or by a protracted immersion in alcohol or acetone, soak it for several hours in a rather heavy chloro-resin solution, then try to break the enamel with a chisel or by grinding with a coarse stone. An old tooth which has been dried out for years, and the enamel of which is as brittle as glass, can be made as tough as a fresh tooth in a very few hours by this simple process. This is an excellent way to treat your technic teeth so as to prevent the chipping off of the enamel while you are preparing them for various uses and also to prevent their cracking and checking in after years.

Of course, if this solution will carry sufficient resin into the inter-prismatic spaces to have this effect on old, dried-out teeth, there can be no argument about its carrying sufficient resin into the infinitely larger tubuli to effectually obliterate them and encapsulate in its dense, solid subsance any bacteria they contain. Even granting (as some contend) that the resin is not a germicide or antiseptic, a bacterium encapsulated in it has about as much chance to functionate and proliferate as the proverbial snowball has in the southern seaport town usually mentioned in connection with it.

Before I terminate this part of my paper, I wish to refute a statement contained in an article which appeared in the September *Cosmos,* to the effect that a pulp-canal filling made by the Callahan method could not be shown by the X-ray. This is totally erroneous, as will be shown by every one of my illustrations. The writer states: ''The radiogram showed the filling as far as the cone penetrated, but did not show where the solution of gutta-percha had filled the canal.'' If this Callahan filling had been properly made there would have been nothing but a heavy ''doughy'' solution of gutta-percha in the canal—no cone left. That there is a great difference in the radio-shadowgraph of an extracted tooth and one in situ I admit, but laboratory findings must always be correlated to clinical findings.—*Journal N. D. A.,* for February, 1921.

